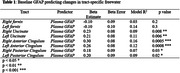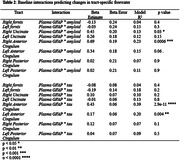# Plasma GFAP, cortical amyloid, and cortical tau interact to predict neuroinflammatory changes within Alzheimer’s‐relevant white matter bundles

**DOI:** 10.1002/alz.095354

**Published:** 2025-01-09

**Authors:** Brandon J Hall, Nesrine Rahmouni, Arnaud Boré, Seyyed Ali Hosseini, Joseph Therriault, Arthur C. Macedo, Lydia Trudel, Tevy Chan, Firoza Z Lussier, Yi‐Ting Wang, Stijn Servaes, Etienne Aumont, Jaime Fernandez Arias, Yansheng Zheng, Cécile Tissot, Jenna Stevenson, Tharick A. Pascoal, Nicholas J. Ashton, Henrik Zetterberg, Kaj Blennow, Maxime Descoteaux, Pedro Rosa‐Neto

**Affiliations:** ^1^ Translational Neuroimaging Laboratory, The McGill University Research Centre for Studies in Aging, Montréal, QC Canada; ^2^ Université de Sherbrooke, Sherbrooke, QC Canada; ^3^ University of Pittsburgh, Pittsburgh, PA USA; ^4^ Lawrence Berkeley National Laboratory, Berkeley, CA USA; ^5^ Departments of Psychiatry and Neurology, University of Pittsburgh School of Medicine, Pittsburgh, PA USA; ^6^ Department of Psychiatry and Neurochemistry, Institute of Neuroscience and Physiology, The Sahlgrenska Academy, University of Gothenburg, Mölndal, Gothenburg Sweden; ^7^ Institute of Neuroscience and Physiology, Sahlgrenska Academy at the University of Gothenburg, Gothenburg Sweden; ^8^ Institute of Neuroscience and Physiology, Sahlgrenska Academy at the University of Gothenburg, Göteborg Sweden; ^9^ McGill University Research Centre for Studies in Aging, Douglas Research Centre, Montreal, QC Canada; ^10^ McGill University, Montreal, QC, Canada, Montréal, QC Canada; ^11^ Douglas Mental Health University Institute, Montreal, QC Canada

## Abstract

**Background:**

The presence of cortical amyloid‐beta pathology is associated with white matter microstructural changes in Alzheimer’s disease (AD), especially in tracts associated with memory. However, the relationships between tract‐specific neuroinflammation and plasma markers of astrogliosis is underexamined; similarly, the involvement of tau neurofibrillary tangles is unclear in neuroinflammation. Here, we investigated the association between plasma glial fibrillary acidic protein (GFAP) and changing proportion of intravoxel freewater—a microstructural change associated with neuroinflammation—within white matter tracts vulnerable to AD.

**Method:**

We sampled 182 subjects from the TRIAD cohort with baseline plasma GFAP, apoe genotyping, ^18^F‐NAV4694 (amyloid) and ^18^F‐MK6240 (tau) PET scans, and both baseline and longitudinal diffusion MRI data (110 female; age 69.8±6.6 years; 64 apoe4 carriers; 70 amyloid positive; 38 tau positive; time difference 60.2±18.8 weeks). dMRI was processed with Tractoflow, freewater_flow, rbx_flow to extract intravoxel freewater in the uncinate fasciculus, the anterior cingulum, the posterior cingulum, and fornix (https://github.com/scilus/freewater_flow). Linear regressions tested the main effect of GFAP on change in tract‐specific freewater. Interaction tests examined the effects between a) amyloid positivity b) tau PET and GFAP on freewater. Model covariates: age, sex, and apoe genotype. T‐tests examined group differences in GFAP. ^18^F‐NAV4694 positivity was assessed with a standardized uptake value ratio (SUVR) versus cerebellar gray matter. ^18^F‐MK6240 SUVR was calculated in a meta‐ROI versus inferior cerebellar grey matter. Statistics performed in R 4.0.3.

**Result:**

We observed that elevated baseline GFAP predicts increases in freewater in the anterior cingulum, posterior cingulum, and uncinate fasciculus (**Table 1**). The interaction between GFAP and amyloid status predicted greater freewater accumulation in the uncinate fasciculus and anterior cingulum; this was seen between GFAP and tau only in the anterior cingulum (**Table 2**). Baseline GFAP was higher in the amyloid positive and tau positive participants (both p < 0.00001).

**Conclusion:**

Freewater in the uncinate fasciculus, anterior cingulum, and posterior cingulum increased in participants with elevated baseline GFAP, which indicates that this plasma marker of astrogliosis correlates with increased neuroinflammation. Moreover, there is an interaction between GFAP and both amyloid and tau PET in the anterior cingulum, which suggests that both proteinopathies contribute to neuroinflammation in this tract.